# Crystal Gold

**Published:** 1859-10

**Authors:** 


### CRYSTAL GOLD.

A few years ago much was written and said upon the Crystal Gold subject. There was much discussion upon the point of its efficiency and durability. Considerable said for it, and much more against it ; this was probably a natural result, for taking the mass of operations, the failures were far more numerous than the successes. This might with truth be said of some other things than crystal gold. In a matter of this kind, the failures do not prove the unfitness or inefficiency of the material in question. But the success does prove both its fitness and efficiency. Five hundred men may try to make a gimlet of steel, and fail, and come to the sage conclusion that gimlets can't be made of steel, while one man who understands the matter, makes a perfect gimlet in a few minutes, and is able to demonstrate that good gimlets can be made of nothing else than steel.

We design to make a simple statement in regard to crystal gold fillings, and we do so because the statement has been so frequently and unhesitatingly made, that crystal gold fillings will become disintegrated, and fail to accomplish the object for which they were made.

This day, Sept. 12, Mrs. H. called to have her teeth examined. Two years and five months previous to this time, I filled seventeen cavities with crystal gold; eight of these fillings were in the anterior teeth, and these extensively decayed; to-day, these seventeen fillings are in as perfect condition as when made; there is no disintegration, the plugs are just as solid as when they were put in, they retain their finish perfectly, and not one of the teeth is in the slightest degree discolored. There has been no sensitiveness of the dentine since they were filled, though there was considerable previous, and at the time of filling.

In this case, the structure of the teeth was of an imperfect character, soft and decaying rapidly.

We speak of this case, because it was such an unfavorable one in the outstart, and the success is so signal. We might refer to hundreds of other cases, equally as successful, both our own operations and those of others. The finest fillings we have ever seen were made of crystal gold. We do not wish to revive the crystal gold controversy, but we do intend from time to time to speak of the success with, and efficiency of this much abused article; for we do believe as firmly as ever that perfectly crystalized gold, well manipulated, makes more perfect fillings than gold in any other form. T.

